# Interaction networks for identifying coupled molecular processes in microbial communities

**DOI:** 10.1186/s13040-015-0054-4

**Published:** 2015-07-15

**Authors:** Magnus Bosse, Alexander Heuwieser, Andreas Heinzel, Ivan Nancucheo, Hivana Melo Barbosa Dall’Agnol, Arno Lukas, George Tzotzos, Bernd Mayer

**Affiliations:** 1 Emergentec Biodevelopment GmbH, Gersthoferstrasse 29-31, 1180 Vienna, Austria; 2 Vale Institute of Technology, Rua Boaventura da Silva, 955. Nazaré, Belém, Pará Brazil

**Keywords:** Network biology, Microbial cooperation, Bioleaching, Chalcopyrite, Acidithiobacillus, Emergence

## Abstract

**Background:**

Microbial communities adapt to environmental conditions for optimizing metabolic flux. Such adaption may include cooperative mechanisms eventually resulting in phenotypic observables as emergent properties that cannot be attributed to an individual species alone. Understanding the molecular basis of cross-species cooperation adds to utilization of microbial communities in industrial applications including metal bioleaching and bioremediation processes. With significant advancements in metagenomics the composition of microbial communities became amenable for integrative analysis on the level of entangled molecular processes involving more than one species, in turn offering a data matrix for analyzing the molecular basis of cooperative phenomena.

**Methods:**

We present an analysis framework aligned with a dynamical hierarchies concept for unraveling emergent properties in microbial communities, and exemplify this approach for a co-culture setting of At. ferrooxidans and At. thiooxidans. This minimum microbial community demonstrates a significant increase in bioleaching efficiency compared to the activity of individual species, involving mechanisms of the thiosulfate, the polysulfide and the iron oxidation pathway.

**Results:**

Populating gene-centric data structures holding rich functional annotation and interaction information allows deriving network models at the functional level coupling energy production and transport processes of both microbial species. Applying a network segmentation approach on the interaction network of ortholog genes covering energy production and transport proposes a set of specific molecular processes of relevance in bioleaching. The resulting molecular process model essentially involves functionalities such as iron oxidation, nitrogen metabolism and proton transport, complemented by sulfur oxidation and nitrogen metabolism, as well as a set of ion transporter functionalities. At. ferrooxidans-specific genes embedded in the molecular model representation hold gene functions supportive for ammonia utilization as well as for biofilm formation, resembling key elements for effective chalcopyrite bioleaching as emergent property in the co-culture situation.

**Conclusions:**

Analyzing the entangled molecular processes of a microbial community on the level of segmented, gene-centric interaction networks allows identification of core molecular processes and functionalities adding to our mechanistic understanding of emergent properties of microbial consortia.

## Background

Microorganisms populate their environment not necessarily as solitude metabolic entities but may form metabolic communities comprising of several species. Such microbial communities are under synergistic and cooperative control with further constraints imposed by environmental factors. Under certain conditions such communities may exhibit emergent properties, i.e. phenotypic observables not being attributable to any single species but only to the community as such. The observation of mutual dependence of some species on the services and/or products of other community members is widely observed, but in many cases hampers comprehensive investigation at the level of an integrated molecular process analysis [1].

Metagenomics as core technology for species identification [2], further combined with omics on the transcript, protein and metabolite level allow to study molecular superpathways as a composite of the entire underlying molecular network contributed by each individual species. Such combined omics has provided unprecedented insights into the functioning, ecology and evolution of microbial communities [3–5]. Proper handling and integrative analysis of such molecular data space characterizing microbial communities has allowed embedding phenotypic observables into various theoretical frameworks as the evolutionary selection for cooperation [6], the public goods dilemma in consortia [7], or avoidance of evolutionary traps [8].

Further experimental advancements have boosted resolution of microbial community research, including procedures offering improved sensitivity to capture also low-abundant species [9], or an approach for allowing profiling novel microorganisms without being limited by gene/protein data available in reference sequences, in consequence significantly expanding the scope of metagenomics research [10]. Together, such technologies have popularized metagenomics techniques finding applications in diverse areas such as human health, nutrition, industrial production, or environment remediation [11].

A number of industrial processes are based on utilization of microbial consortia. One prominent example is extraction of base metals from mineral ores, termed bioleaching, where the potential of bacteria-enabled biotechnologies has been successfully demonstrated [12–14]. In such systems, consortia of bacteria enhance the rate of metal recovery from ores containing valuable metals in the form of mineral sulfides, e.g. sphalerite (ZnS) or chalcopyrite (CuFeS_2_), or for recovery of gold encapsulated in pyrite (FeS_2_). The communities are composed of specialized, mostly mesoacidophilic and chemolithotrophic bacteria. Community composition is dynamic in response to changes in temperature, pH, concentration of ferric iron or toxic metals, or salinity [15].

Extraction of the metal from sulfide occurs spontaneously by cleavage of the mineral’s Fe-S_2_ bond (through ferric ions, oxidative attack) as well as via protons (hydrolytic attack) according to the general reactions [16]:

1 $$ \mathrm{M}\mathrm{S}+\mathrm{F}{\mathrm{e}}^{3+}+2\ {\mathrm{H}}^{+}\ \to\ {\mathrm{M}}^{2+}+0.5\ {\mathrm{H}}_2{\mathrm{S}}_{\mathrm{n}}+\mathrm{F}{\mathrm{e}}^{2+}\left(\mathrm{n}\ \ge\ 2\right) $$

2 $$ 0.5\ {\mathrm{H}}_2{\mathrm{S}}_{\mathrm{n}}+\mathrm{F}{\mathrm{e}}^{3+}\ \to\ {\mathrm{S}}_0+\mathrm{F}{\mathrm{e}}^{2+}+{\mathrm{H}}^{+} $$

Bioleaching bacteria are not directly involved in mobilization of metals from minerals via an enzymatic process. Rather, they accelerate metal solubilization indirectly by providing ferric ions (iron oxidation; (3)) as well as protons (sulfur oxidation; (4)) [17]:

3 $$ 4\ \mathrm{F}{\mathrm{e}}^{2+}+{\mathrm{O}}_2+4\ {\mathrm{H}}^{+}\ \to\ 4\ \mathrm{F}{\mathrm{e}}^{3+}+2\ {\mathrm{H}}_2\mathrm{O} $$

4 $$ 2\ {\mathrm{S}}_8+3\ {\mathrm{O}}_2+2\ {\mathrm{H}}_2\mathrm{O}\ \to\ 2\ \mathrm{S}{{\mathrm{O}}_4}^{2-}+4\ {\mathrm{H}}^{+} $$

At an industrial scale, bioleaching systems are used in recovery of metal ores (e.g. gold and silver) or to leach base metals such as copper, cobalt, nickel and zinc, as well as low-grade uranium ores. Extending the approach to non-base metals is not yet commercially attractive. The main reasons for the lack of commercial bioleaching operations are seen in efficiency constraints, costs associated with the construction of suitable bioreactors as well as in logistic issues related to vast volume of material to be processed [13]. Recently, mining companies are confronted with a set of drivers that affect the economics of mining. Among those is a growing global demand for raw metals as well as tightening environmental legislation. Efficiency gains can be expected through the adoption and/or optimization of bioleaching processes [17].

Metagenomic data sets generated during the last decade have massively expanded the knowledge base on bioleaching consortia. First level analyses mainly focused on determining community composition and species population dynamics. Further analysis aimed at understanding key metabolic pathways involved in energy production, fixation of carbon and nitrogen, or bacterial survival in highly acidic and/or toxic environments [12, 18–20].

Still, from a systems biology perspective it is desirable to go beyond a parts-based approach and focus on cooperation from an entangled molecular process point of view [12, 21]. This approach is particularly important in view of lacking efficient genetic engineering platforms for major bioleaching microbes next to regulatory constraints [12]. One promising option for achieving a more comprehensive understanding of collaborative functionalities is to approach bioleaching consortia like single ‘superorganisms’. For executing such approach, data are frequently organized in a network context, resembling molecular features as nodes and molecular interactions as edges. Such networks then undergo functional category- or topology-based analysis aimed at identifying molecular processes associated with phenotypic readouts of interest (utilized in various systems biology examples e.g. in human health [22, 23]). Functional insights at the molecular process level are to be expected if metadata on the various omics levels are integrated in a way that allows the linkage of specific community molecular processes to phenotypic observables becoming apparent only at the superorganism level. Such approach represents a shift from a species/individual molecular component point of view towards structuring of information across species boundaries. This approach retrieves molecular processes integrating molecular features from more than one species, by this providing molecular process models underlying cooperative phenomena.

From a formal perspective such phenomena have been described by Baas et al. as dynamical hierarchies [24], already applied in diverse chemical [25] and biological systems [26]. The concept starts with first order objects (e.g. protein coding genes), based on interaction forming higher order structures (functional categories, pathways), which in turn exhibit properties not identified on any lower order level. As practical example serves biofilm formation, if combined further with thiosulfate, the polysulfide and the iron oxidation pathways resulting in yet another phenotypic readout, or emergent property as efficient leaching of minerals. Still, specific functional properties may be contributed from individual species of a microbial community, and a network representation of community functional properties in contrast to analysis on the level of individual species is deemed necessary for identification of community properties.

Romo et al. [27] described a bioleaching system which (i) exhibits an emergent community effect in terms of mineral leaching efficiency, (ii) is composed of a minimum number of species which (iii) have rich annotation at the gene and protein level. The authors investigated the efficiency of chalcopyrite (CuFeS_2_) bioleaching in a controlled setting with pure cultures of Gram-negative gamma-proteobacteria Acidithiobacillus ferrooxidans and At. thiooxidans compared to a co-culture of both bacteria, where the latter about doubled recovery rate of copper. Similar observations were reported in studies conducted by Fu et al. [28] and – albeit in an overall much less efficient system, by Qui et al. [29]. A striking observation with these minimal systems is the fact that one of the species, At. ferrooxidans, alone possesses both sulfur and iron oxidizing properties deemed essential for bioleaching [13, 30]. Yet, a co-culture with At. thiooxidans, a sulfur- but not iron-oxidizer [31], results in a significant increase in leaching rate, raising the question about the nature of the cooperation of the two microbes.

In this work we implement a network biology methodology resting on functional annotation and interaction information of the protein coding gene space of both organisms aimed at identification of cooperation with significant increase in bioleaching as emergent property. We exemplify the approach on core molecular processes executed by At. thiooxidans and At. ferrooxidans in chalcopyrite bioleaching.

## Methods

### Gene annotation

Gene-centric annotation on a species level utilized publicly available databases. As primary data source for annotation of At. ferrooxidans (ATCC 53993) and At. thiooxidans (ATCC 19377) the Pathosystems Resource Integration Center (PATRIC, [32]) with the respective PATRIC organism identifiers 6930 and 52187 was used. Further data retrieval utilized the Integrated Microbial Genomes database (IMG, [33]) using IMG taxon object identifiers 642788233 and 2510461056 for At. ferrooxidans and At. thiooxidans, respectively. In addition to gene annotation sourced from PATRIC and IMG, data from the Expasy family of gene/protein resources (HAMAP, [34]; Prosite [35]; PSORT [36]) as well as the EBI-EMBL resources InterPro [37] and UniProt [38] were obtained using the online tools provided on the respective sites.

According to PATRIC At. ferrooxidans holds 2,872 protein coding genes, the number for At. thiooxidans is 3,076. Mapping of annotation across PATRIC and IMG identifiers was done automatically for At. ferrooxidans using the UniProt ID mapping tool [39], and due to minor coverage provided by UniProt via manual assignment for At. thiooxidans. Gene-centric annotation details harvested from the various sources are provided in Table 1A.

**Table 1:** Gene annotation and interaction details

Annotation item	source	gene coverage, At. thiooxidans	gene coverage, At. ferrooxidans
A: gene annotation			
**Gene/protein identifiers**			
PATRIC database ID	PATRIC	100.00	100.00
RefSeq gene ID	PATRIC	100.00	100.00
Uniprot gene name	PATRIC	88.62	91.43
IMG database ID	IMG	88.62	54.18
RefSeq protein ID	PATRIC	0.00	91.43
Uniprot ID	UniProt	0.97	83.88
**Protein name & function**			
Protein name	PATRIC	100.00	100.00
Protein function	HAMAP	26.17	29.84
Enzyme Commission nomenclature	PATRIC	17.55	19.74
IMG TERM	IMG	19.90	25.90
COG category	IMG	69.25	70.40
COG entry	IMG	69.25	70.40
**Protein domains/families**			
Prosite domain	Prosite	30.92	33.50
FIGfam protein family ID	PATRIC	66.29	99.02
Pfam protein family	IMG	72.79	77.61
Protein similarity (HAMAP)	HAMAP	28.41	33.04
**Protein function & location**			
Interpro database entry	InterPro	4.48	33.11
GO function	HAMAP	3.71	29.07
GO process	HAMAP	25.68	29.49
GO cellular component	HAMAP	17.65	20.26
PSORT subcellular location	PSORT	100.00	100.00
**Pathway assignment**			
KEGG pathway	PATRIC	17.56	19.74
KEGG orthology	IMG	44.90	51.36
KEGG module	IMG	17.13	20.44
Metacyc pathway	IMG	17.04	19.50
IMG pathway	IMG	7.87	9.96
B: interaction information			
pathway relations	KEGG	13.00*	16.47
pathway relations	Metacyc	11.05*	12.99
inferred interactions	STRING	51.07*	91.16

Annotation on gene (**A**) and interaction level (**B**) for At. ferrooxidans and At. thiooxidans. Data sources are grouped in content categories. Annotation coverage refers to the percentage of protein coding genes holding a valid entry in the respective data sections for gene annotation, and at least one interaction to another gene as derived from interaction sources. *For At. thiooxidans interactions were assigned based on ortholog assignment

Interaction annotation data (Table 1B) were sourced from STRING [40], MetaCyc [41] and KEGG [42]. MetaCyc interactions were inferred based on co-membership in MetaCyc pathways restricted to genes holding an Enzyme Commission number [43]. To obtain KEGG interaction data KGML+ files containing KEGG pathway maps for At. ferrooxidans were downloaded from the KEGG website, and interactions were extracted using the Bioconductor KEGGraph package [44]. STRING interaction data (At. ferrooxidans) were directly retrieved from the STRING web server. As no interaction data source hosts specific data for At. thiooxidans, interactions were assigned to At. thiooxidans genes based on ortholog mapping to At. ferrooxidans utilizing the Needle algorithm from the EMBOSS package [45]. In case of multiple assignments the top scoring ortholog was selected. In total 1,661 ortholog genes are identified for the two species, allowing the imputation of interactions for the subset of orthologs identified on the At. thiooxidans side.

Further annotation specifically in the context of microbial consortia and bioleaching was performed via mining of NCBI Pubmed [46]. A keyword catalog was applied for covering scientific publications on cooperative aspects (synergism, consortium, community, cooperation) and specific utilization (bioleaching, biomining, chalcopyrite). The query term included the gene name together with a respective catalog entry.

### Analysis procedures

Gene annotation and interaction information as provided in Table 1 allows designing a gene-centric data structure for each of the At. ferrooxidans and At. thiooxidans genes using the PATRIC ID as unique structure identifier. The core data structure represents the protein coding gene annotation of both organisms, and further defines aggregate nodes by e.g. grouping genes according to gene assignment to clusters of orthologous groups (COG) [47]. By this the set of gene-centric data structures is traversed into a set of COG-based data structures.

**Fig. 1:**
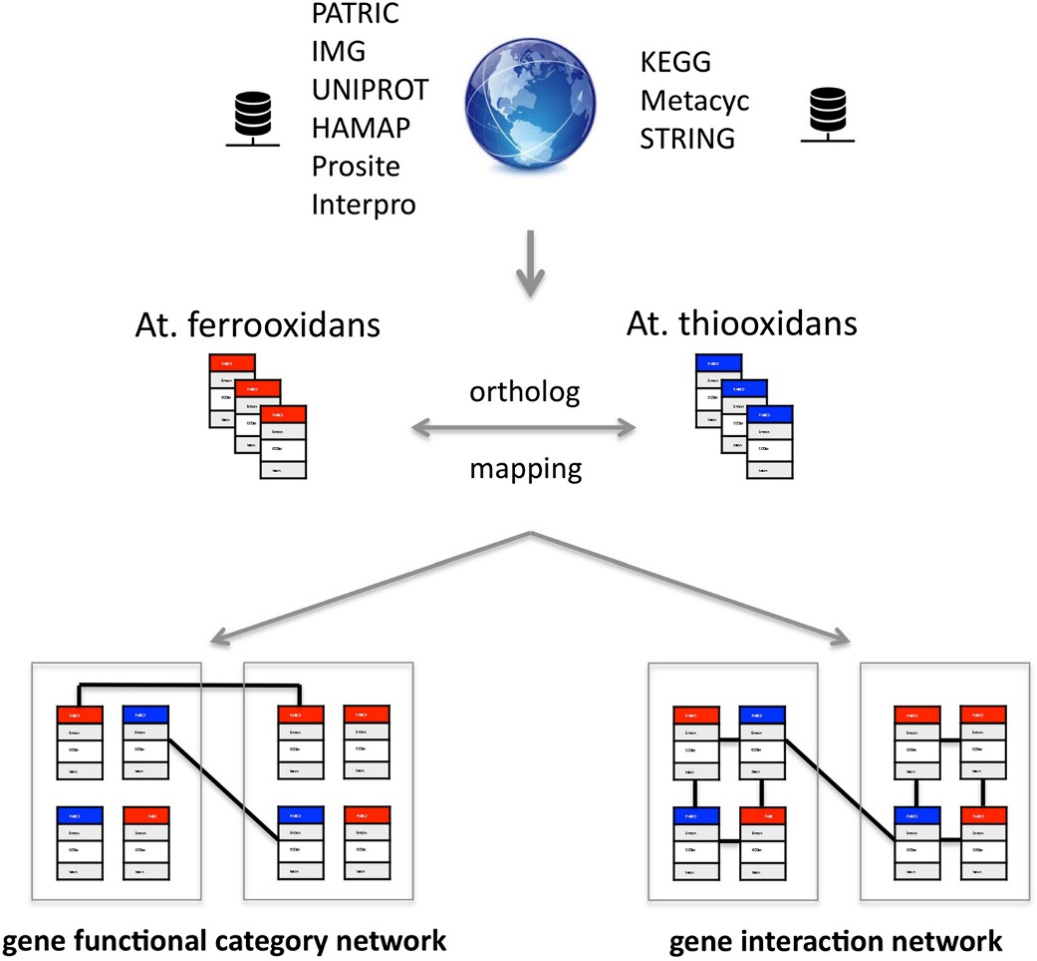
From annotation data to interaction networks. Public domain repositories are utilized for gene-centric annotation as well as for retrieving protein coding gene interaction information. Data structures holding the PATRIC ID as central identified are populated with annotation and interaction information, including ortholog mapping for adding to comprehensiveness in annotation. Cross-species interaction networks are built deductively resting on gene functional category assignments (as COG terms) and inductively by utilizing gene interaction information

On the protein coding gene level, as well as on an aggregate level of COG terms interaction networks can be derived (Fig. 1).

On the level of COG terms such networks represent co-annotation of genes in more than one COG term. Reciprocal assignment of genes to two COG terms infers functional closeness of such terms, expressed by an interaction. On a gene level interaction networks are derived by making use of gene interaction information. First, a network is built including all ortholog genes together with interaction information according to annotation. For identifying network segments with high inner connectivity segmentation was performed using MCODE with default settings [48]. With a given network structure as start MCODE identifies most densely connected subgraphs, from there expanding to neighborhood network nodes with a cutoff criterion for stopping expansion according to the connectivity of such neighboring nodes. Specifically, starting with a given set of genes (input set) and given interaction information the induced subgraph is derived, eliminating all genes with a connectivity of zero. Network segments (units) are derived from this induced graph via MCODE, where each such unit is interpreted as functional context (molecular process). Utilizing interaction information of features across identified segments allows approximating functional dependencies between molecular processes, finally leading to a molecular process model representation of the input gene set. For segmentation and visualization of networks the MCODE plugin of Cytoscape was used (version 3.1.1, [49]).

## Results

### Dynamical hierarchies in microbial communities

**Fig. 2:**
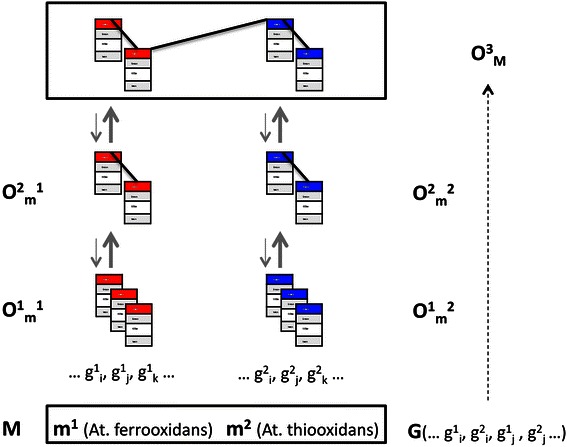
Formal representation of a microbial community. Starting with a microbial community **M** composed of individual species **m**^**i**^ and the set of protein coding genes **G** observables become apparent on the individual gene level (**O**^**1**^), on the pathway level for each species (**O**^**2**^), and on the level of inter-species molecular processes finally generating a community observable **O**^**3**^. Integrative analysis aims at deriving a model on the level of individual genes **g**^**i**^ explaining an emergent property **O**^**3**^_**M**_

According to annotation on gene and interaction level a data structure is populated for each protein coding gene of At. ferrooxidans and At. thiooxidans. Each structure encodes different levels of hierarchy, namely each gene as first order object (further characterized by a property space including among others domain and function details), as well as aggregate assignments on the level of gene ontology, pathway and COG terms. Such aggregate (higher order) objects are per construction resulting of the properties of first order elements together with interactions. Assignment of a first order object to specific higher order terms reduces the degrees of freedom of the first order object, i.e. forming a dynamical hierarchy exhibiting up- and downward causalities (Fig. 2).

On this basis we assume explicit encoding of molecular functional properties of the microbial co-culture **M** (a set of microbial species **m**^**i**^). In addition, we detect a significant bioleaching efficiency increase in the co-culture situation, which in the following is denoted as observable **O**^**3**^_**M**_. Such observable rests on properties of individual genes (**O**^**1**^) and properties of aggregate states observed on the level of molecular pathways (**O**^**2**^). Our analysis goal is identification of the set of first order objects, being the protein coding genes **g**^**i**^, in synthesis (interactome) leading to the phenotypic readout **O**^**3**^_**M**_. As in our specific scenario such readout is observable in the specific co-culture situation we assume involvement of first order objects (protein coding genes) from both, At. ferrooxidans and At. thiooxidans.

We know **O**^**3**^_**M**_**:= M**, being a mapping of the set of two species (or *n* species in a general metagenomics setting). This mapping does not reveal any type of context structure, hence no functional **f(M)** allowing us to model **O**^**3**^_**M**_. This status is equivalent to the first result of a metagenomics analysis, i.e. the list of distinct species involved. **M** is a composition of a set of individual species **m**^**i**^**,** where each **m**^**i**^ itself exhibits observables, **O**^**2**^_**m**_^**i**^, i.e. a readout of a species-specific pathway. Such observables are the consequence of the total set of molecular components **g**^**i**^_**j**_ of **m**^**i**^. Describing **O**^**2**^_**m**_^**i**^ as **f(g**^**i**^_**j**_**)** having just a context-free listing of **g**^**j**^_**i**_ provides no better system representation, as we end up with the mapping **O**^**2**^_**m**_^**i**^**:= g**^**i**^_**j**_. When including interactions for allowing modeling of pathways such observable may reflect the capacity of At. ferrooxidans to oxidize both sulfur and iron, whereas At. thiooxidans shows sulfur oxidation only.

We further see **G** as the total set of molecular components of **M**. With **G** we break up the boundaries to individual **m**^**i**^, and obtain a mapping of **O**^**3**^_**M**_ := **G**, which allows describing the community observable as a function of the community genome [50]. Consequently, the observable of interest results from a functional **f**(**G**), where **G** is the superset of molecular constituents of both organisms, **g**^**i**^_**j**_, and when adding interaction information **G** becomes the superpathway. We postulate that the molecular process leading to our emergent property of interest is identified at the level of such superpathway.

According to our goal definition we seek **O**^**3**^_**M**_**:= f(g**_**i**_**)** out of **G**. Such **(g**_**i**_**)** denotes a defined subset of **G** in a specific context, in a biological interpretation being individual molecular processes **p**^**i**^ out of the total set of processes **P**. Consequently, the phenotype **O**^**3**^_**M**_ of interest results from a functional **f**(**P**). Making use of the data structures centered around genes **g**^**i**^_**j**_ specific **p**^**i**^ leading to **O**^**3**^_**M**_ may be derived on some aggregate level, e.g. COG categories resembling **O**^**2**^_**m**_^**i**^, or may be derived bottom up on the level of **G**.

### Bioleaching model

**Fig. 3:**
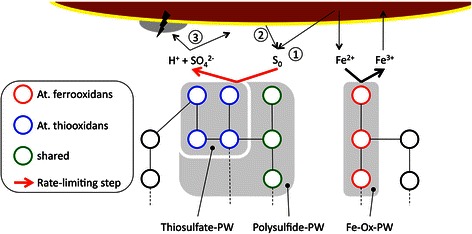
Pathway-centric bioleaching model. Pathway model for cooperation of At. ferrooxidans and At. thiooxidans in chalcopyrite bioleaching. Species-specific molecular processes **p**^**i**^ (schematic subgraphs represent protein coding genes and interactions) assigned to the thiosulfate, the polysulfide and the iron oxidation pathway cooperate in mineral dissolution from chalcopyrite. Mechanisms deemed responsible for increased leaching efficiency in a co-culture setting at the interface with the mineral surface as indicated by arrows include (1) proton availability, (2) sulfur layer removal, (3) hindering jarosite formation

Background knowledge proposes key molecular pathways of each individual species, At. ferrooxidans and At. thiooxidans involved in chalcopyrite leaching. Such given process information on the level of **O**^**2**^_**m**_^**i**^ identifies a principal mechanism for chalcopyrite leaching (Fig. 3). A main cooperative effect is located in the oxidative recycling of ferrous iron and elemental sulfur, respectively [16]. While iron oxidation is only mediated by At. ferrooxidans, sulfur oxidation is a capability shared by both species. Two such pathways are identified, the Polysulfide pathway seen in both Acidithiobacilli, and a Thiosulfide pathway unique to At. thiooxidans [30, 31, 51]. This additional sulfur oxidation pathway is believed to address a leaching bottleneck by increasing the oxidation rate of sulfur, thereby (i) providing additional protons to attack the mineral directly [52], (ii) removing an inhibitory sulfur layer precipitating at the mineral surface [53] and (iii) reducing ambient pH low enough to prevent formation of jarosite, a basic hydrous sulfate of potassium and iron [28, 54]. Jarosite is known to reduce At. ferrooxidans bioleaching efficiency [55].

Molecular processes embedded in pathway nomenclature allow deriving a model adding to understanding of improved bioleaching efficiency in the co-culture situation. However, such annotation is species-specific, i.e. on the level of **O**^**2**^_**m**_^**i**^, hence not providing an integrated representation of collaboration at a molecular process level.

### Coupled molecular processes, aggregate mode

**Fig. 4:**
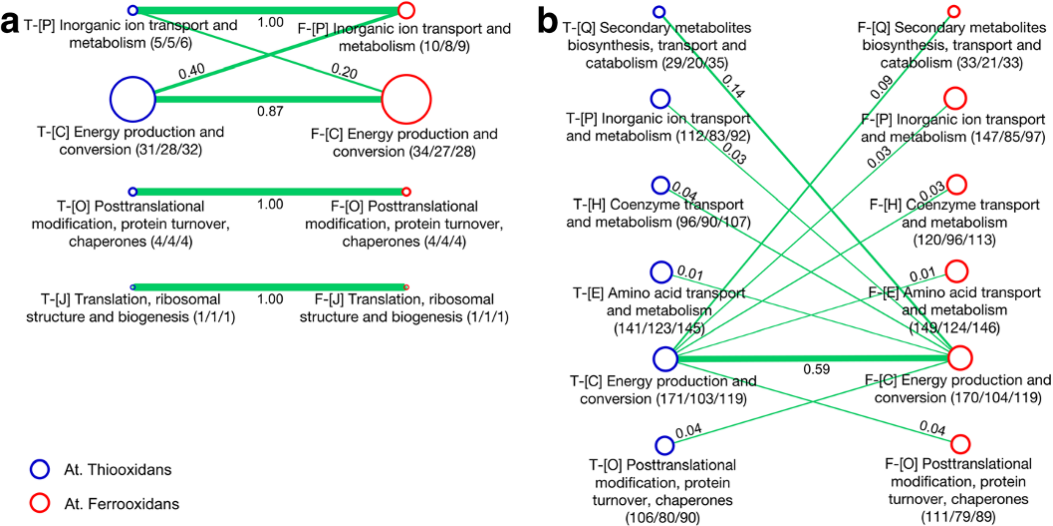
Comparative species analysis, COG level. **a** COG categories (nodes) relevant in At. ferrooxidans (red) and At. thiooxidans (blue) according to thiosulfate, polysulfide and iron oxidation pathway assignment. Each node holds COG category name, number of genes assigned, number of genes also holding orthologs in the respective COG for the other species, and total number of orthologs, i.e. also indicating multiple ortholog assignments. Edge scaling and numbers represent the percentage of ortholog genes calculated normalized to the species holding the lower number of genes in the respective category. **b** graph construction as in (A), but the ortholog network is based on the entire gene sets of both species. The graph focuses on the COG category Energy production and conversion and further COG categories holding orthologs

For obtaining a representation of core molecular processes according to Fig. 3 across species boundaries an aggregate representation, e.g. at the level of COG terms, can be used. Such approach further takes advantage of increased annotation coverage compared to a pathway representation (see Table 1 for functional category versus specific pathway coverage). Assigning genes included in the thiosulfate (9 genes of At. thiooxidans), polysulfide (both species, 11 genes) and iron oxidation pathway (At. ferrooxidans, 18 genes) to COG categories maps to four such categories, namely inorganic ion transport and metabolism, energy production and conversion, posttranslational modification and translation, and ribosomal structure and biogenesis (Fig. 4a).

The COG category holding the highest number of non-ortholog genes is energy production and conversion, with 28 such genes on the At. ferrooxidans side, and 32 genes with At. thiooxidans, essentially resembling specific functions of iron oxidation and thiosulfate pathway.

Expanding ortholog screening for the core COG category energy production and conversion (with in total 289 genes for At. ferrooxidans and 290 genes for At. thiooxidans) to all COG categories identifies five additional COG terms holding orthologs at varying degree (Fig. 4b), including secondary metabolite biosynthesis, inorganic ion transport, coenzyme transport, amino acid transport, and posttranslational modification. Ortholog assignment results in linking of specific transport-associated functionalities which may well add to the cooperative phenomenon as outlined in Fig. 3. Such additional categories hold further a number of species-specific genes, with in total 221 genes on the At. ferrooxidans side, and 151 genes for At. thiooxidans. For deciphering if members of this gene set prone to being involved in key aspects of chalcopyrite leaching processes as presented in Fig. 3 are already identified in the cooperation context or in the specific bioleaching utilization context keyword-base literature screening was performed, with results presented in Table 2.

**Table 2:** COG terms and annotation details in microbial cooperation and bioleaching

COG term	At. ferrooxidans			At. thiooxidans		
	#genes	cooperation	utilization	#genes	cooperation	utilization
(C) Energy production & conversion	66	7	3	68	6	1
(E) Amino acid transport & metabolism	25	4	0	18	2	0
(H) Coenzyme transport & metabolism	24	0	0	6	0	0
(O) Posttranslational modification, protein turnover & chaperones	32	0	0	26	0	0
(P) Inorganic ion transport & metabolism	62	0	0	29	0	0
(Q) Secondary metabolites biosynthesis, transport & catabolism	12	0	0	9	0	0

Number of species-specific genes for At. ferrooxidans and At. thiooxidans regarding COG terms coupling on an ortholog level with the COG term energy production and conversion, and number of such genes reported in scientific literature in the context of cooperation (synergism, consortium, community, cooperation) or specific utilization (leaching, bioleaching, chalcopyrite)

A fraction of non-ortholog genes are reported in the context of cooperative function, and to a lesser degree also in the very specific context of bioleaching. In the COG term energy production and conversion, both Acidithiobacilli hold *Cytochrome B561* and *NADH dehydrogenase* in respect to cooperation. At. ferrooxidans further reports *Aldo-keto reductase*, *Ferredoxin reductase*, *Ferredoxin-like protein*, *Ferredoxin* and *Aldehyde dehydrogenase*. In the specific context of bioleaching *Cytochrome C4* and *Hydroxylamine reductase* are found. In respect to COG term amino acid transport & metabolism *Serine acetyltransferase*, *Homocitrate synthase*, *Cysteine desulfurase* and *Chorismate mutase* record for cooperation. For At. thiooxidans *Acetate kinase*, *Glycerol kinase*, *Oxidoreductase* list for the COG term energy production and conversion in a cooperative context, the latter protein also in a specific utilization context. For the COG term amino acid transport and metabolism *Glutamate decarboxylase* and *Transglutaminase-like protein* are found in the context of cooperation.

### Coupled molecular processes, first order mode

Leaching of chalcopyrite involves as main term energy production and conversion, on a pathway level expressed as thiosulfate, the polysulfide and the iron oxidation pathway being attributable on the individual species level **m**_**i**_. Exploring the involved gene sets on an aggregate mode of COG terms identifies via ortholog mapping further potential mechanisms of relevance in cooperative effects, specifically transport of e.g. coenzymes and amino acids. Ortholog analysis identified a set of species-specific genes **g**_**i**_, to some extend already holding annotation in the context of collaborative effects and bioleaching. According to our concept we aim for identifying **O**^**3**^_**M**_**:= f(g**_**i**_**)** out of **G**, where such **g**_**i**_ constitute a molecular process **p**^**i**^ seeing contributions from both species.

Focusing on the COG term energy production and conversion, additionally taking transport categories into account (secondary metabolites biosynthesis, transport and catabolism; inorganic ion transport and metabolism; coenzyme transport and metabolism; amino acid transport and metabolism) results in 386 ortholog genes of At. ferrooxidans and At. thiooxidans, and 195 genes specifically with At. ferrooxidans and 168 genes specifically with At. thiooxidans. Utilizing interaction data from STRING an ortholog network can be derived, i.e. a graph with nodes encoding the ortholog genes and edges representing the gene-specific interaction information. This ortholog graph holds 385 genes (1 gene of the input gene set had no single interaction information in STRING and is omitted from further analysis) and an Index of Aggregation of 1.0, i.e. each node holds a path to all other nodes of the network.

**Fig. 5:**
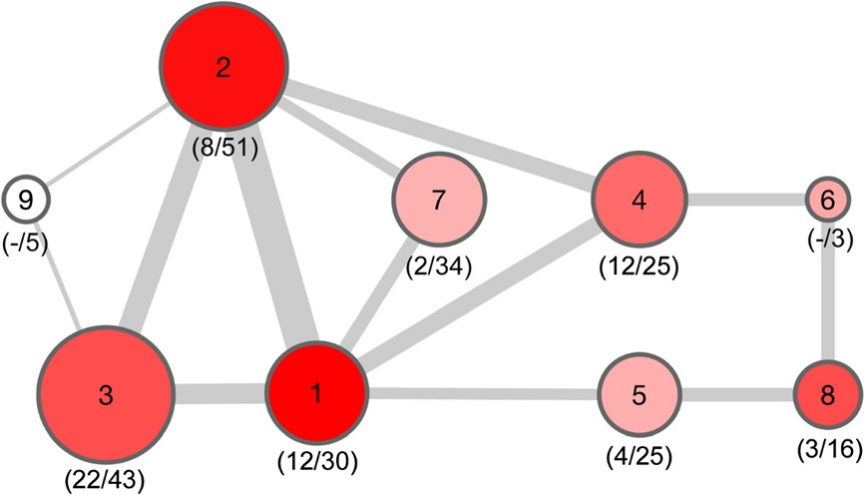
Molecular model of cooperation. Ortholog molecular model on energy production and conversion together with selected transport categories. Nodes represent molecular units, with the node diameter scaling with the number of features included. Edges across units indicate significant dependencies of molecular features across units. Color-coding represents the number of interactions of At. ferrooxidans-specific genes linkable to ortholog genes embedded in units. Numbers in brackets below each node indicate genes assigned to energy production and conversion and number of genes assigned to transport categories (secondary metabolites biosynthesis, transport and catabolism; inorganic ion transport and metabolism; coenzyme transport and metabolism; amino acid transport and metabolism)

Forwarding this induced subgraph to MCODE segmentation results in 9 molecular units, with the largest unit holding 65 genes, the smallest 3. In total 295 genes of the input feature set are part of the unit set. Using STRING interaction information, an aggregate interaction can be computed across units scaling with the sum of interactions of individual unit members to members of other units, thereby generating a molecular process model. STRING further provides specific interaction information for At. ferrooxidans, allowing linking non-ortholog genes to features embedded in units, hence adding specific functionality to the molecular model (Fig. 5).

We consider the molecular units presented in Fig. 5 as individual molecular processes **p**^**i**^ sharing genes across species boundaries, allowing us to postulate a mapping of **O**^**3**^_**M**_ := **f**(**p**^**i**^). In the given analysis the ortholog model involving COG terms energy production and conversion together with specific transport terms is used as core structure, adding molecular functionality coming from At. ferrooxidans only (lack of At. thiooxidans-specific interaction data).

Of specific interest are molecular processes integrating energy production and conversion and transport terms, hence, specifically molecular process units 1, 3 and 4. Unit 1 aggregates genes associated with proton transport, nitrogen metabolism and iron oxidation (e.g. *NADH ubiquinone oxidoreductase* and *Glutamate synthase*). Generally enriched in energy production is unit 3 hosting several *Cytochrome O ubiquinol oxidases*, *ATP synthases* as well as dehydrogenases such as *Malate*, *Isocitrate* and *Pyruvate dehydrogenases*. Functional links to sulfur oxidation is provided in unit 4 (*Rhodanese-like protein*, *Cytochrome D* and *O ubiquinol odixases*). Unit 6 exclusively covers aspects of nitrogen metabolism (*Nitrogen regulating protein P-II*, *Ammonium transporter*). In the context of bioleaching, metal ion transporters are of particular importance. Those are mainly embedded in unit 2 (magnesium and cobalt transport proteins as well as lead, cadmium, zinc and copper transport ATPases) and unit 7 (zinc transporters, ferric iron uptake regulators, ferrous iron transporters). Given interaction data result in multiple assignments of genes to process units. One major representative seeing such multiple assignments is *Carbonic anhydrase*, an enzyme involved in the sulfur cycle.

## Discussion

Integrative analysis on the grounds of omics data promises improved characterization of molecular processes mediated by microbial communities. Of particular interest are processes involving direct dependencies across species boundaries resembling a major driver for exhibiting emergent properties. A formal concept allowing representation of such phenomena is dynamical hierarchies, which are composed of individual objects holding a data structure encoding properties and interactions. In a composition individual objects form higher order structures which may direct to emergent properties (phenotypic observables) that are not amenable at any lower level hierarchy, and specifically not reducible to a single object. In the context of molecular systems this concept can be applied to sets of genes as first order objects, potentially leading to an emergent property based on a molecular process, which in turn is composed of a specific set of genes. This concept is directly applicable to microbial communities showing synergistic readout, with the example of significantly increased mineral leaching in a co-culture of At. ferrooxidans and At. thiooxidans.

A necessary prerequisite for implementing such approach is rich gene annotation covering from domain and function assignment to aggregate properties as functional category or pathway membership. A second prerequisite is interaction information implicating dependencies for executing molecular processes, which in combination with gene annotation allows setting up a data structure for building interaction networks on the gene level or any aggregate level as COG terms.

Utilizing species-specific pathway information allows deriving a first mechanistic model aimed at describing phenomena adding to improved bioleaching in the co-culture situation, essentially involving the thiosulfate, the polysulfide and the iron oxidation pathway. These constituents improve mineral dissolution from chalcopyrite via proton attack, sulfur layer removal, and hindrance of jarosite formation. Analyzing involved genes on the level of COG terms and ortholog mapping links the central term energy production and conversion with specific transport processes, namely secondary metabolites biosynthesis, transport and catabolism, inorganic ion transport and metabolism, coenzyme transport and metabolism, and amino acid transport and metabolism. This term set includes a number of non-ortholog genes from both species, a subset being already discussed in the context of microbial communities and bioleaching.

Certainly, a description at the level of COG terms does not allow delineation of integrated molecular processes. Building gene-centric interaction networks followed by segmentation according to topological criteria provides means for approximating such processes. Executing this procedure for the ortholog network of At. ferrooxidans and At. thiooxidans with focus on energy production and conversion coupled with identified transport terms results in a molecular process model bridging energy production and transport processes in shared molecular units. Integrated processes identified include iron oxidation, nitrogen metabolism and proton transport, segments covering sulfur oxidation and nitrogen metabolism, and a set of ion transporter functionalities. Including At. ferrooxidans-specific genes in this network results in a composite representation of shared community functionality and specific add-ons of one species. Of particular interest is *Carbonic anhydrase*, described as a constituent to the polysulfide pathway [56]. This gene has been found as associated with biofilm formation in a very recent proteomics study [57]. While the exact contributions of *Carbonic anhydrase* to a bioleaching process are unknown, three mechanisms can be conceived, including (i) positive impact on ammonia utilization of other consortia members by removal of environmental carbon disulfide (CS_2_) [58], (ii) enabling At. ferrooxidans to utilize CS_2_ as alternative energy source and (iii) contributing to inorganic carbon fixation [59]. A second gene, *Homocitrate synthase*, is described as an essential component of lysine biosynthesis and has been found upregulated in a screen searching for quorum-sensing-related molecules [60], suggesting a role in cell-density-dependent processes implicated in the formation of biofilms.

Taken together, the additional genes contributed by At. ferrooxidans enable a cornerstone role in the establishment and perpetuation of a chalcopyrite leaching consortium. This is consistent with the results of a recently conducted metabolomic study using a laboratory community composed of At. thiooxidans and ferrooxidans strains. This study identified biofilm formation via At. ferrooxidans, suggesting a central role in community establishment, as consequence fostering increased bioleaching activity as emergent property [61].

## Conclusions

A set of genes is identified on a common mechanistic background of At. ferrooxidans and At. thiooxidans, as these are components of molecular processes delineated from the ortholog network covering energy production and conversion together with transport processes. Recalling the concept of dynamical hierarchies such processes provide an integrated perspective of the superpathway composed of the individual constituents of both species together with their interactions.

The approach presented in this work allows deriving hypotheses on coupled molecular processes, providing an alternative perspective of synergistic effects and community-based emergent properties. With the concept of dynamical hierarchies established, availability of annotation and interaction information in the public domain, and open source libraries for computing networks and segments this approach is of general applicability. A major present pitfall is consolidated availability of molecular data needed to populate the data structures. Necessary gene annotation is distributed across numerous individual sources, hampering integration for given annotation beyond the challenge of adding annotation to novel species identified in metagenomics. Utilizing orthology for annotation imputation naturally results in biases, becoming even more pronounced when going to a larger set of species composing the community. Of equal relevance, and with even less coverage presents interaction information, being either generic on a pathway level, or only covering selected species. For fully leveraging on the power of integrative, network-based analysis procedures realizing common grounds on annotation and interaction information represents a major challenge ahead in the field, partly addressed by hybrid approaches in approximating interactions as done with STRING.

Delineation of coupled molecular processes associated with emergent properties, as synergistic increase in bioleaching efficiency, offers improved understanding of microbial community dynamics. Further, such molecular process knowledge provides the basis for selection of molecular biomarker candidates indicative for such community molecular function. Biomarkers in their definition serve as proxy for monitoring the status of a molecular process, in a microbial community needing to capture molecular processes established across individual species. Segmented, cross-species interaction networks provide coupled process sets as basis for selection of biomarker candidate panels. Such panels in turn can be utilized as tool for screening microbial communities prone to exhibiting an emergent property of interest.
